# The complete chloroplast genome of an economic plant, *Chrysanthemum morifolium* ‘Baekma’

**DOI:** 10.1080/23802359.2019.1666682

**Published:** 2019-09-23

**Authors:** Swati Tyagi, Jae-A Jung, Jung Sun Kim, Soo-Jin Kwon, So Youn Won

**Affiliations:** a Genomics Division, National Institute of Agricultural Sciences, Rural Development Administration, Jeonju, Republic of Korea;; b Floriculture Research Division, National Institute of Horticultural and Herbal Science, Rural Development Administration, Wanju, Republic of Korea

**Keywords:** Asteraceae, *Chrysanthemum*, chloroplast genome

## Abstract

*Chrysanthemum morifolium* (*Dendranthema grandiflorum*), known as florist’s daisy is an important ornamental and medicinal plant of the Asteraceae family. The complete chloroplast genome sequence of one economic cultivar ‘Baekma’ was 151,060 bp in length with a large single copy (LSC) region (82,862 bp), a small single copy (SSC) region (18,294 bp) and two inverted repeats (IRs) (24,952 bp). It contained 130 genes, including 85 protein-coding genes, 8 rRNAs and 37 tRNAs. The overall GC content was 37%. Phylogenetic analysis showed that *C. morifolium* ‘Baekma’ was grouped together with other *Chrysanthemum* species.

*Chrysanthemum morifolium* (*Dendranthema grandiflorum*), an ornamental and medicinal plant in the family Asteraceae, is important in the global floricultural industry. Many cultivars of *C*. *morifolium* showing the diverse flower shape, size and colors have been developed through the long history of breeding. The cultivar ‘Baekma’, a standard type with a single large and white flower per stem, is a significant commodity in the cut flower market including Korea and Japan, and also serves as a genetic resource for breeding (Lee and Lee 2015). Apart from ornamental purposes, *C. morifolium* has been reported to treat various brain, liver and kidney-related disease in traditional folk medicines (Gui et al. 2014). The complete chloroplast genome of *C. morifolium* ‘Baekma’ will help us to identify, protect and utilize this particular cultivar and to further understand genetic diversity and evolution process within the genus *Chrysanthemum* and family Asteraceae.

*Chrysanthemum morifolium* ‘Baekma’ was developed by the National Institute of Horticultural and Herbal Science and registered as IT232551 in the National Agrobiodiversity Center (Jeonju, Korea; N 35°49′53.8″, E 127°03′45.4″) (Shin et al. 2005). The total genomic DNA was extracted from fresh leaves and subjected for library preparation and sequencing by Illumina’s HiSeq platform (California, USA). The complete chloroplast genome was determined by the *de novo* assembly of low coverage whole-genome shotgun (WGS) sequences dnaLCW (Kim et al. 2015) and annotated using online program Dual Organellar GenoMe Annotator (DOGMA) (http://dogma.ccbb.utexas.edu/) with manual corrections (Wyman et al. 2004). The complete genome sequence *C. morifolium* ‘Baekma’ together with gene annotations were submitted to NCBI GenBank under the accession number MK986830.

The chloroplast genome of *C. morifolium* ‘Baekma’ was 151,060 bp in length, which was composed of a large single copy (82,862 bp) and a small single copy (18,294 bp) regions separated by a pair of inverted repeats (24,952 bp). The overall GC content of the genome was 37%. Since all ribosomal RNA (rRNA) genes with high GC composition were located in IR regions, the GC content of IR region was higher (43%) than SSC (31%) and LSC (36%). A total of 130 functional genes were annotated including 85 protein-coding genes, eight rRNAs and 37 tRNAs, in addition to one pseudogene. The number of intron-containing genes was 15; 13 genes with one intron, two genes with two introns and one was trans-spliced. Genes located in the IR regions, including four rRNAs were found to be duplicated, which was commonly observed in other Asteraceae species (Zhang et al. 2016).

Phylogenetic analysis was performed by aligning all of the chloroplast genome using ClustalΩ program and *Nicotiana tabacum* (Solanaceae) as an outgroup (Sievers et al. 2011). The maximum likelihood tree indicated that *C. morifolium* ‘Baekma’ was clustered with other *Chrysanthemum* and Asteraceae species (Figure 1). Based on the chloroplast sequence of *C. morifolium* ‘Baekma’, molecular markers can be designed to distinguish this economic cultivar from others and evolutionary and phylogenetic studies can be conducted.

**Figure 1.**
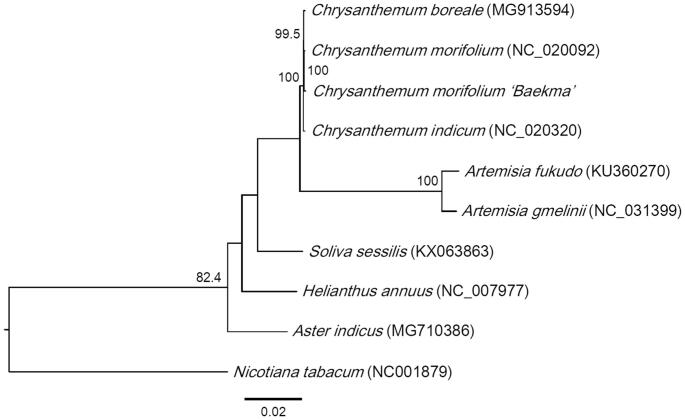
Phylogeny of *Chrysanthemum morifolium* ‘Baekma’, other Asteraceae species and *Nicotiana tabacum*. Maximum-likelihood tree was constructed with complete chloroplast genome sequences and 1000 bootstrap replicates. The scale bar is substitutions per site.
